# Fungal biotransformation of okara into functional food: Comparative species-dependent metabolomic and antioxidant profiles

**DOI:** 10.1016/j.crfs.2026.101396

**Published:** 2026-04-01

**Authors:** Analdi Farniga, Praphan Pinsirodom, Songsak Wattanachaisaereekul

**Affiliations:** School of Food Industry, King Mongkut's Institute of Technology Ladkrabang, 1 Chalong krung Rd., Ladkrabang, Bangkok, 10520, Thailand

**Keywords:** *Aspergillus*, Fungi, Metabolite, *Neurospora*, Okara, *Rhizopus*, Solid-state fermentation

## Abstract

Okara, a nutrient-dense by-product of soybean processing, is abundantly produced in many Asian countries and holds significant potential for multiple purposes. Solid-state fungal fermentation represents a promising approach to improve its nutritional quality and functional properties; however, there is currently no research available on how fungal starter selection shapes the metabolites in the final product. This study aimed to investigate and compare the metabolite profiles of okara fermented using five different fungi: three species of *Rhizopus* (*Rhizopus microsporus, Rhizopus oligosporus*, and *Rhizopus oryzae*), *Aspergillus oryzae*, and *Neurospora sitophila*. Increased glucan content, which represents the fungal biomass, was observed after all fermentation periods (24 h for *Rhizopus* spp.; 48 h for *A. oryzae*; 72 h for *N. sitophila*). The highest glucan content, at 9.07% w/w, was achieved after fermentation with *N. sitophila*, representing an 11.20-fold increase, with α-glucan accounting for 6.38%. Meanwhile, *R. microsporus* yielded the highest β-glucan content at 5.93%, achieving a 20.45-fold increase. The LC-MS results showed that each starter facilitated the expression of different metabolites. A total of 181 metabolites were detected, with *Rhizopus* spp. notably producing 49–61 unique metabolites, while *A. oryzae* and *N. sitophila* distinctively resulted in formation of betaine and L-glutamic acid. In addition, fungal-fermented okara exhibited elevation of phenolic compounds by 2.19- to 4.13-fold and correspondingly possessed superior antioxidant properties compared to non-fermented okara. All told, the present study provides substantial information on considerations in fungal starter selection for fermenting okara.

## Highlights

• Fungal fermentation of okara increases phenolic content and antioxidant activity.
• Different fungal starters generate unique metabolite profiles.
• *Neurospora sitophila* enhances protein and antioxidant levels most effectively.
• After fermentation, choline content decreases while ergocalciferol increases.
• Chemometric tools reveal metabolite shifts based on the fungal species used.

## Graphical abstract

**Figure:**
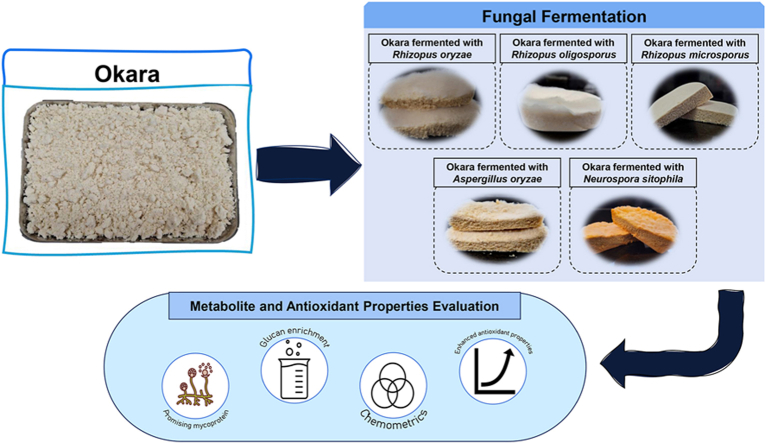

## 1 Introduction

Sustainability is the future currency of human life. The 2030 Agenda for Sustainable Development, unanimously adopted by United Nations Member States in 2015, offers a universal roadmap for achieving peace and prosperity for all people and the planet, both now and in the future. As it stands, our current dietary habits and production methods are causing harm to both land and water ecosystems, leading to depletion of water resources and accelerating climate change while trying to provide for the global population of 8.2 billion people (Foley et al., 2011; Godfray et al., 2010). Re-adjusting our dietary habits is a key element in solving this issue. Studies have shown that compared to the equivalent amount of animal-based food, plant-based foods are lower in CO_2_ emissions, including the usage of land and water (Poore and Nemecek, 2018). Soybean is one of the best sources of alternative protein, and is mainly used in beverages and processed food such as soy milk and tofu. In Thailand, demand for soy milk is trending upward; as of 2022, it reportedly accounts for 30–40% of the total UHT milk market (Prasertsri, 2022). Notably, during soy milk manufacturing, 1.2 kg of wet-solid waste is produced (dos Santos et al., 2019). This solid by-product is known by many names, such as okara, ‘*biji’*, ‘*tofukasu’*, and soybean dreg (Feng et al., 2021).

Though labelled a by-product, okara has high nutritional value, being mainly composed of fibre, protein, and fat (dos Santos et al., 2019). Despite this value, utilisation of okara remains a challenge, with its main usage being only for animal feed (Vong and Liu, 2016). Interestingly, Indonesia has pioneered the transformation of okara from waste to edible food through its biological modification by fungi via solid-state fermentation. Several fungi are used for this purpose. The fungal-fermented okara (FFO) produced by *Rhizopus* spp. is known as ‘*tempeh gembus*’ or ‘*black oncom*’, whereas that produced with *Neurospora* is known as ‘*red oncom*’ (Ginting et al., 2024; Nurmilah et al., 2024). Notably, tempeh and oncom are staple foods that have been consumed in Indonesia for centuries, and are known to provide health benefits (Ahnan-Winarno et al., 2021; Afifah et al., 2015).

Fungi in general play numerous vital roles in the food industry, including as sources of mycoproteins, food ingredients, and nutraceuticals. They can be cultivated in a short period of time and on diverse substrates due to their ability to contextually produce diverse enzymes for biomass hydrolysis. These enzymes, such as amylase (Balakrishnan et al., 2021), cellulase (Farniga et al., 2023), laccase (Chen et al., 2016), lipase (Yu et al., 2016), and protease (Patil et al., 2019), break down complex carbohydrates, fats, and proteins in the substrate, making them more digestible and contributing to the unique flavour and texture of the final product. Correspondingly, fungal starter selection is a critical factor for ensuring the consistency of fermented soybean products, including FFO. Inconsistency in phenolic compound formation has previously been reported in tempeh production (Zhang et al., 2022; Polanowska et al., 2020).

Several studies have applied metabolomics to fermented soy products such as tempeh and soy sauce to identify fermentation-associated changes in bioactive compounds and flavour-related metabolites (Lee et al., 2021; Zhu et al., 2022; Astawan et al., 2025). However, these studies often focus on detailing the activity of a single microorganism; our understanding of the comparative effects of different fungal species remains lacking. The present study characterises the distinct metabolite profiles resulting from the fermentation of okara with five different fungal starters (*Rhizopus microsporus*, *Rhizopus oligosporus*, *Rhizopus oryzae*, *Aspergillus oryzae*, and *Neurospora sitophila*), aiming to provide a comprehensive understanding of how starter selection influences the functional properties of FFO. First, the compatibility of okara as a substrate for the examined fungi was evaluated in terms of glucan production. After that, the samples were analysed to determine the expressed metabolites. Finally, the antioxidant properties of okara and FFO were assayed and compared.

## 2 Materials and methods

### 2.1 Fungi starter preparation

The study utilised five distinct fungi from the School of Food Industry KMITL culture collection, comprising three species of *Rhizopus* (*Rhizopus microsporus* TISTR 3531, *Rhizopus oligosporus* TISTR 3527, and *Rhizopus oryzae* TISTR 3336), *Neurospora sitophila* NRRL 2884, and *Aspergillus oryzae* FI-21 isolated from koji rice. Each fungus was grown on a potato dextrose agar plate for 2–5 days, after which a spore suspension was prepared by harvesting the mycelia using 10 mL of sterile 0.1% (v/v) Tween 80. Spore concentration was determined by means of a Neubauer haemocytometer (Hirschmann Laborgeräte, Germany) under a microscope.

### 2.2 Fungal-fermented okara preparation

**Fig. 1:**
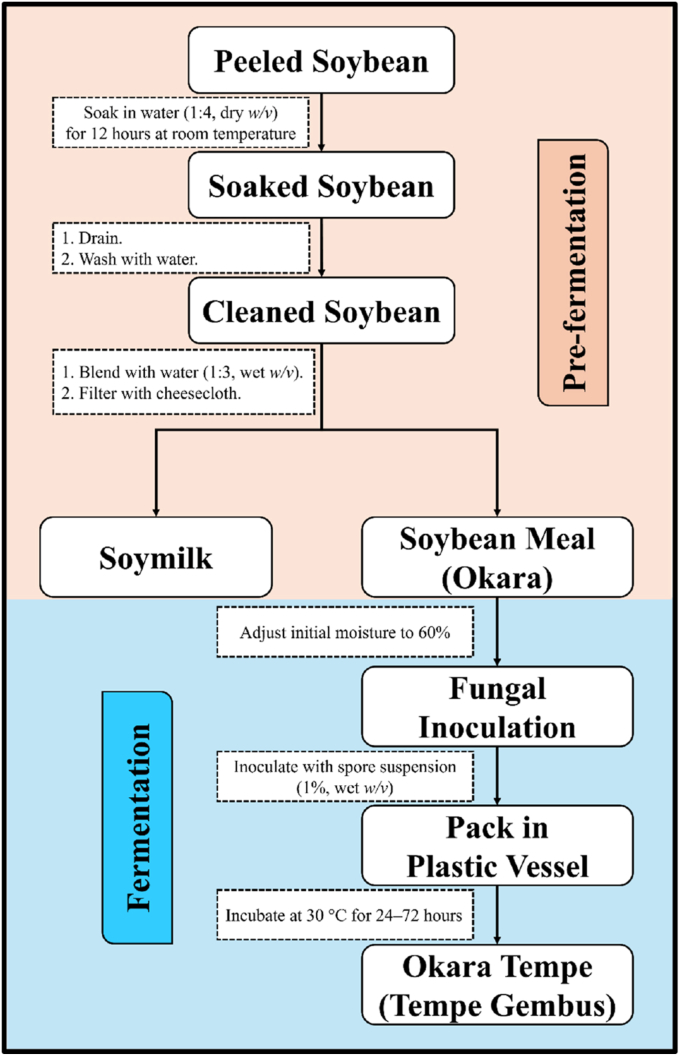
Summary of the process for producing fungal-fermented okara (FFO).

Production of fungal-fermented okara (FFO) began with the preparation of okara from soybean, which was then fermented with each of the prepared fungi cultures as outlined in Fig. 1. First, peeled split soybeans were soaked in water (1:4, w/v) at room temperature for 12 h, then cleaned and blended with water (1:3, w/v). The resulting mixture was filtered with a cheesecloth, and the solid residue (okara) was subjected to the same blending and filtering steps twice more. Finally, the prepared okara was dried in a hot air oven at 60 °C for 12 h. For temporary storage, the okara was kept at 4 °C.

**Fig. 2:**
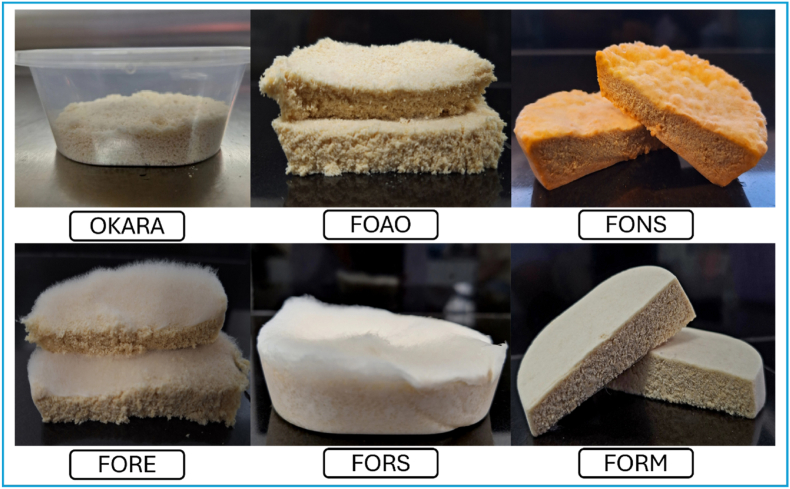
Images illustrating morphological characteristics of the analysed FFO. FORE, fermented okara with *Rhizopus oryzae*; FORS, with *Rhizopus oligosporus*; FORM, with *Rhizopus microsporus*; FOAO, with *Aspergillus oryzae*; FONS, with *Neurospora sitophila*.

FFO was produced aseptically in the microbiology laboratory using a modification of the procedure of Rizal et al. (2024). Prior to fermentation, the moisture content of the okara was adjusted to ∼60% and the material was sterilised in an autoclave at 121 °C for 15 min. FFO was then prepared by inoculating the okara with 1% (w/v) of the appropriate fungus, packing it in a sterile perforated plastic container, and fermenting at 30 °C. The concentrations of the spore suspensions were 1 × 10^6^ for *Rhizopus* spp., 1 × 10^7^ for *A. oryzae,* and the initial prepared spore suspension (undetermined concentration) for *N. sitophila*. Fermentation was conducted over a period of 96 h, with fungal growth monitored at 24-h intervals to evaluate the progression and morphological characteristics of FFO. This time-course evaluation was aimed at identifying the optimal fermentation duration required to achieve the preferred physical and morphological characteristics, as defined by the Indonesian National Standard for soybean tempeh (SNI 3144:2015), which reflects typical consumption standards in Indonesia (see Fig. 2). This standard states that the final product must exhibit a compact texture (not be easily broken), a uniform colour over the entire surface, and an absence of ammonia odour. Based on the observations, the optimal fermentation durations were determined to be 24 h for *Rhizopus* spp., 48 h for *A. oryzae*, and 72 h for *N. sitophila*. These specific fermentation times were subsequently used for the metabolite and antioxidant analyses. All analyses were conducted using three biological replicates.

### 2.3 Glucan analysis

For glucan analysis, okara and FFO samples were first dried in a hot air oven at 60 °C for 12 h and then finely ground. Total glucan, α-glucan, and β-glucan were quantified using the β-Glucan Assay Kit for Yeast and Mushroom (Megazyme, Wicklow, Ireland).

### 2.4 Proximate analysis

Proximate analysis of the samples was conducted in accordance with AOAC International methods to determine moisture, protein, fat, ash, and total carbohydrate contents, and expressed as percentages on a wet basis (Horwitz, 2006). Moisture and ash contents were quantified using gravimetric method. Protein content was determined by Kjeldahl method, using a nitrogen-to-protein conversion factor of 5.71, while fat content was measured by Soxhlet extraction with petroleum ether. Total carbohydrate content was calculated by difference, subtracting the sum of moisture, ash, lipid, and protein contents from 100%.

### 2.5 Metabolites extraction

Fresh samples were extracted using the method of Gu et al. (2019) with modification. Briefly, 10 g of each sample (∼60% moisture content) was mixed with 50 mL of HPLC-grade absolute methanol and homogenised with a hand-mixer. The extraction was then performed in a shaking incubator at 150 rpm (4 °C) for 12 h in dark conditions. Afterwards, the mixture was centrifuged at 10,000×*g* for 10 min and the supernatant collected. For analyses, extracts were diluted with methanol at an appropriate ratio depending on the analysis.

### 2.6 Untargeted metabolomics analysis

The untargeted metabolomics analysis employed ultra-high performance liquid chromatography coupled with electrospray-ionisation triple quadrupole time-of-flight mass spectrometry (UHPLC-ESI-QTOF-MS) (Bruker, Germany). The UHPLC instrument was equipped with a C18 reverse-phase column (imChem, France). Mobile phase **A** consisted of water +2.5 mM ammonium formate buffer, while mobile phase **B** consisted of methanol +2.5 mM ammonium formate buffer. The gradient began with a ratio of 99.0 A:0.1 B from 0 to 10.00 min, shifted to 0.1:99.9 from 10.00 to 12.10 min, and returned to 99.9:0.1 from 12.10 to 15.00 min. MS was performed using the positive ionisation mode and an electrospray voltage of 500 V with the capillary voltage set to 4500 V. The system was configured with a dry gas flow rate of 10.0 L min^−1^ at 220 °C. Spectra were acquired at a rate of 5 Hz and covered the mass range of 20 to 1300 m/z. Compound name, retention time, molecular formula, and molecular mass (m/z) were annotated using the MetaboScape® software with the Bruker MetaboBASE® Personal Library 3.0. Putative annotations were assigned by matching experimental MS/MS spectra to the library using a similarity score threshold of >60. As the individual annotated metabolites are not confirmed by the reference standards, all metabolites were classified as putatively annotated compounds according to the Metabolomics Standards Initiative (MSI) confidence levels 2–3.

### 2.7 Data processing of LC-MS analysis

Annotated metabolites obtained from UHPLC-ESI-QTOF-MS were categorised according to their compound groups, identified with reference to the open-access databases ChemSpider (https://www.chemspider.com/), FooDB (https://foodb.ca/), the Kyoto Encyclopedia of Genes and Genomes Pathway Database (https://www.genome.jp/kegg/), LIPID MAPS (https://lipidmaps.org), Pubchem (https://pubchem.ncbi.nlm.nih.gov//), and The Human Metabolome Database (https://hmdb.ca/). Metabolites were trimmed and converted to percentage concentrations by dividing the area of each metabolite by the total area of all detected metabolites. Chemometrics analysis was then carried out using the MetaboAnalyst 6.0. platform (Pang et al., 2024).

Prior to chemometrics analysis, data were log-transformed, mean-centred, and auto-scaled (divided by the standard deviation of each variable). Principal component analysis (PCA) was then conducted to develop a preliminary understanding of the overall metabolite profiles. Afterwards, partial least squares-discriminant analysis (PLS-DA) was performed to elucidate further differences. Metabolites having a variable importance in projection (VIP) score >1 were considered metabolite markers and were selected for heatmap clustering analysis. Overlap among profiles was also visualised with Venn diagrams generated in VENNY 2.1 (https://bioinfogp.cnb.csic.es/tools/venny/index.html).

### 2.8 Antioxidant, total flavonoid, total phenolic acid, and total condensed tannin assays

Antioxidant compound content and activity assays were conducted in 96-well plates based on the spectrophotometric methods described in our previous publication (Puspitasari et al., 2024). Absorbances were read by a microplate reader (PerkinElmer Inc., USA). All analyses were conducted in three biological replicates. The standard curves used to determine concentrations achieved *R*^2^ > 0.99.

#### 2.8.1 Determination of phenolic compounds

Total phenolic content (TPC) was measured using the method of Bobo-García et al. (2015) with modification. Briefly, 25 μL methanol extract was mixed with 25 μL Folin-Ciocalteu reagent and diluted with 200 μL water. The mixture was incubated for 5 min at room temperature, after which 25 μL of 10% (w/v) sodium carbonate was added, and the mixture incubated for another 60 min at 25 °C. Finally, the absorbance was read at 765 nm. The standard curve consisted of gallic acid in ethanol at concentrations of 0 to 200 μg mL^−1^.

Total flavonoid content (TFC) was quantified using a reported method by Pękal et al. (Pękal and Pyrzynska, 2014) with modification. Eighty μL of methanol extract was mixed with 80 μL of 2% (w/v) aluminium chloride (in ethanol) and 120 μL of 50 g L^−1^ sodium acetate. This mixture was incubated for 150 min at 25 °C, after which the absorbance was read at 440 nm. The standard curve utilised quercetin in ethanol at concentrations of 0 to 50 μg mL^−1^.

Total condensed tannin content (TCTC) was determined by the method of Price et al. (1978) with modification. Twenty-five μL of methanol extract was mixed with 150 μL of 4% vanillin and 25 μL of sulfuric acid. The mixture was then incubated for 15 min at 25 °C and the absorbance determined at 500 nm. The standard curve consisted of 0 to 1000 μg mL^−1^ catechin in ethanol.

#### 2.8.2 Antioxidant assays

To determine antioxidant capacity, three assays were utilised. The first was the 1,1-diphenyl-2-picryl-hydrazyl (DPPH) assay from Mensor et al. (2001) with modification. Forty μL of extract was combined with 260 μL of DPPH (0.1 mmol L^−1^), incubated for 30 min at 25 °C, and then the absorbance at 517 nm was read. Second was a 2,2′-azinobis-(3-ethylbenzothiazoline-6-sulfonic acid) (ABTS) assay modified from the method reported by Re et al. (1999). First, ABTS^+^ was produced by reacting 5 mL of 7 mmol L^−1^ ABTS solution with 88 μL of 140 mM potassium persulfate in the dark at 4 °C for 12 h. The ABTS^+^ was then diluted by mixing 0.5 mL ABTS^+^ solution with 45 mL ethanol. Prior to analysis, the ABTS^+^ concentration was adjusted to an equivalent absorbance of ∼0.7 (read at 734 nm). For the analysis, 250 μL of diluted ABTS^+^ was mixed with 10 μL of extract, incubated for 6 min at 25 °C, and the absorbance measured at 734 nm. Finally, the ferric reducing antioxidant power (FRAP) assay was utilised according to the method from Benzie et al. (Benzie and Strain, 1996), with modification. Briefly, the reagent was prepared by mixing 300 mmol L^−1^ sodium acetate buffer, 10 mmol L^−1^ 2,4,6-tripytidyl-s-triazine, and 20 mmol L^−1^ ferric chloride at a ratio of 10:1:1. Then, 280 μL of the reagent mixture was mixed with 20 μL of extract and incubated at 37 °C for 10 min. Afterwards, the absorbance was read at 593 nm. For all antioxidant assays, the standard curve was constructed using ascorbic acid in methanol at concentrations of 0 to 500 μg L^−1^.

## 3 Results and discussion

### 3.1 Glucan analysis

Glucan is the main component of the fungal cell wall, and hence an increase in glucan content is directly proportional to fungus proliferation (Ruiz-Herrera and Ortiz-Castellanos, 2019), making it an ideal marker for monitoring fungal growth. Fungal growth can in turn correlate with the fungal fermentation performance. To assess the compatibility of okara as a substrate for fungi, this study determined the total glucan, α-glucan, and β-glucan contents in both unfermented and fermented okara.

**Fig. 3:**
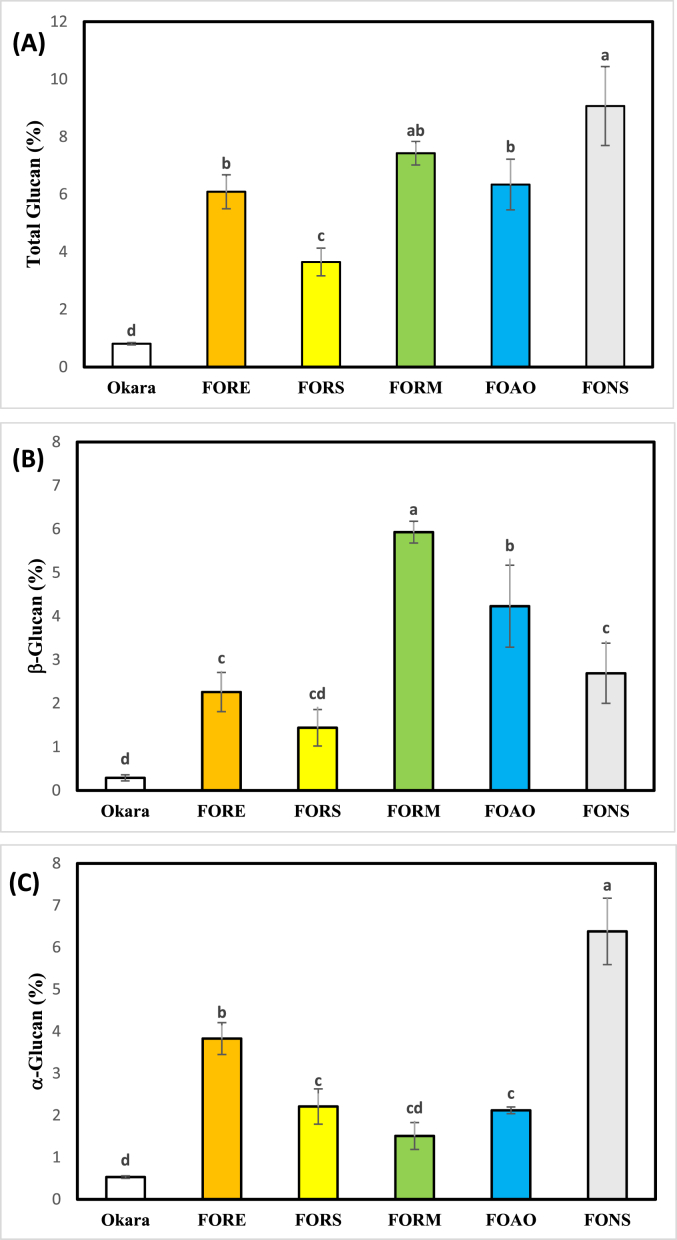
Percentage glucan in FFO. (**A**) Total glucan; (**B**) β-glucan; (**C**) α-glucan. Different letters above bars indicate statistically significant difference in means based on Tukey's HSD at the 95% confidence level.

Increased glucan content was observed after all fermentation periods (24 h for *Rhizopus* spp.; 48 h for *A. oryzae*; 72 h for *N. sitophila*) (Fig. 3), indicating that okara is a compatible substrate for all of the tested strains. This aligns with the findings of Pilafidis et al. (2024), who also reported increased glucan production during fungal fermentation of agricultural by-products. Correspondingly, the significant increase in glucan content across all fungal fermentations was expected, given the ability of the tested fungi to produce a wide range of carbohydrate-active enzymes, lipases, and proteases, which can efficiently break down the components of okara (Corrêa et al., 2014). Indeed, okara is known to be rich in insoluble polysaccharides like pectin and cellulose (Yamaguchi et al., 1996; Sun et al., 2012), which can serve as substrates for fungal growth. Notably, the highest glucan content (reaching 9.07% w/w*,* an 11.20-fold increase) was observed following fermentation with *N. sitophila* fermentation (FONS), which predominantly produced α-glucan (6.38%). This suggests that *N. sitophila* possesses a robust enzymatic system for degrading okara's complex carbohydrates. Meanwhile, the highest β-glucan content (5.93%) was obtained when using *R. microsporus* (FORM), with an impressive 20.45-fold increase.

This highlights the potential of fungal fermentation to not only valorise okara but also enhance its nutritional profile. Especially, the selective enhancement of β-glucans in FORM has significant implications, as β-glucans are known for diverse health benefits, including immunomodulatory and cholesterol-lowering effects (Flores et al., 2024). Antibacterial activity of β-glucans has also been reported in tempe gembus produced by co-culturing *R. oligosporus* and *Saccharomyces cerevisiae* (Rizal et al., 2024). In contrast, the role of α-glucans as indicators of fermentation performance remains poorly understood. It is worth noting that the abundance and structural features of α-glucans in fungal cell walls are known to vary across fungal species and in response to fermentation conditions. Interestingly, α-glucan isolated from *Xylaria* sp. has recently been shown to exhibit antibacterial and antioxidant activities (Wang et al., 2025). Therefore, the high glucan production observed in the present study warrants further investigation, particularly regarding structural elucidation and evaluation of the functional properties of the produced glucans.

### 3.2 Proximate analysis

Analysing the moisture, protein, fat, ash, and carbohydrate content of fermented okara (Table 1) revealed that ash content gradually increased and moisture content decreased as fermentation progressed, with the least moisture and the most ash observed in FONS, at 56.03% and 3.39%, respectively. Increasing mineral content during fermentation is due to loss of dry matter such as carbohydrates, protein, and fats as fungi degrade the substrate (Day and Morawicki, 2018). Fermentation can increase levels of magnesium, iron, calcium, and zinc by decreasing the number of phytates (Pranoto et al., 2013), and furthermore increases bioavailability through the liberation of minerals bound by biomolecules, depending on the duration of the fermentation (Lopez et al., 1983). In addition to the highest ash content, FONS also exhibited the highest concentrations of protein and carbohydrates, achieving statistical significance at *p* < 0.05. Notably, the high protein levels in FONS are consistent with a prior report that fermentation by *Neurospora*’ augments okara protein content (Maini Rekdal et al., 2024). Meanwhile, FONS also exhibited decreased fat contents, which might relate to lipase accumulation over the course of the longer fermentation interval (Arooj et al., 2023), similar to the case of okara fermented with a co-culture of *S. cerevisiae* and *Hansenula* sp., reported by Shi et al. (2020).

**Table 1:** Results of the proximate analysis.

Sample	Moisture	Protein	Fat	Ash	Carbohydrate
**Okara**	59.95 ± 0.13^aA^	15.60 ± 0.05^bB^	12.63 ± 0.25^abC^	2.33 ± 0^dE^	9.48 ± 0.23^abD^
**FORE**	58.92 ± 0.53^aA^	15.23 ± 0.23^bcB^	13.55 ± 1.20^aB^	2.84 ± 0.01^cD^	9.46 ± 1.87^abC^
**FORS**	59.01 ± 2.25^aA^	16.02 ± 0.78^bB^	11.97 ± 1.20^abBC^	2.88 ± 0.04^cD^	10.12 ± 2.85^abC^
**FORM**	59.21 ± 0.49^aA^	14.58 ± 0.04^cB^	14.52 ± 0.79^aB^	2.90 ± 0.11^cD^	8.80 ± 0.78^bC^
**FOAO**	57.35 ± 0.15^abA^	15.70 ± 0.05^bB^	12.71 ± 1.13^abC^	3.06 ± 0.07^bD^	11.18 ± 1.34^abC^
**FONS**	56.03 ± 0.17^bA^	17.27 ± 0.32^aB^	10.13 ± 0.79^bD^	3.39 ± 0.04^aE^	13.18 ± 0.53^aC^

∗FORE, fermented okara with *Rhizopus oryzae*; FORS, with *Rhizopus oligosporus*; FORM, with *Rhizopus microsporus*; FOAO, with *Aspergillus oryzae*; and FONS, with *Neurospora sitophila*.

Values are present in percentages (%) on a wet basis.

Different letters indicate statistically significant difference in means based on Tukey's HSD at the 95% confidence level (lowercase: column; uppercase: row).

Okara fermented by different *Rhizopus* species (FORE, fermented okara with *R. oryzae*; FORS, with *R. oligosporus*; FORM, with *R. microsporus*) was found to differ slightly in carbohydrate, fat, and protein formation. In particular, protein content in FORE and FORS was not significantly different from unfermented okara (*p < *0.05), while a reduction was observed in FORM. Meanwhile, FORM exhibited the highest fat content (14.52%), slightly more than previously reported for ‘*HongJun Tofu’* (okara fermented with *N. crassa*) (Qiu et al., 2022). This variance among *Rhizopus* species is likely attributable to differential enzyme expression (Suresh et al., 2021; De Freitas et al., 2014). In this case, *R. microsporus* might have focused on consuming carbohydrates and protein, leading to an increase in relative fat content. It could also promote retention of lipids within its biomass, such as ergosterol, which is commonly abundant in *Rhizopus* (Zwinkels et al., 2023). More detailed profiling of amino acids and fatty acids can be conducted to better understand carbon preference in fungal feeding (Tahmasian et al., 2023).

### 3.3 Metabolite profiling by LC-MS

**Fig. 4:**
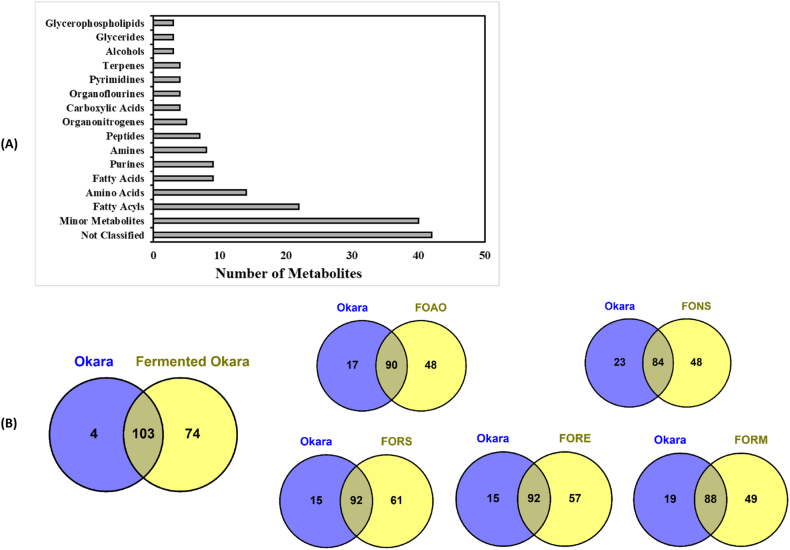
Metabolite profiles of okara and FFO. (**A**) Total metabolites detected by class; (**B**) Venn diagrams showing overlap of total metabolites between samples. Minor metabolites represent the pool of chemical groups with no more than two detected metabolites.

LC-MS identified a total of 181 metabolites across unfermented and fermented okara preparations (Fig. 4). This number is relatively high within the context of existing metabolomic studies on fungal-fermented soybean products, particularly tempeh. For example, a previous GC-MS-based analysis of over-fermented tempeh, in which *R. oligosporus* was used as the starter culture, reported detection of 121 metabolites (Prativi et al., 2023). The overall broader metabolite set observed in the present study may be attributed to the use of multiple fungal species and of specific fermentation conditions optimised for okara substrate. The detected metabolites were classified into 16 groups, with 42 metabolites remaining unannotated based on the databases used in this study (Fig. 4A). The presence of these unclassified metabolites suggests the possibility of novel compounds, highlighting the need for further structural elucidation and investigation into their potential bioactivities. A complete list of the identified metabolites is provided in Supplementary Table S1. Lipid derivatives were prominent, including fatty acids, fatty acyls, glycerides, glycerophospholipids, and prenol lipids, indicating significant lipid metabolism during fermentation. This is consistent with previous research demonstrating fungal capacity to modify lipid profiles in various substrates (Oliveira et al., 2011; Li et al., 2022).

Overall, 103 metabolites (56.9%) were common to both okara and FFO, while 74 (40.9%) were exclusively found in the fermented samples (Fig. 4B). This substantial increase in unique metabolites underscores the profound impact of fungal fermentation on okara's metabolic composition. Furthermore, each of the five fungal species produced distinct metabolite patterns. Notably, FOAO (fermented okara with *A. oryzae*) and FONS (fermented okara with *N. sitophila*) shared 48 unique metabolites, suggesting *A. oryzae* and *N. sitophila* to induce similar metabolic pathways. Meanwhile, the highest numbers of unique metabolites were obtained with *Rhizopus* spp. fermentation, despite the shorter fermentation time (24 h), with 49 in FORM, 57 in FORE, and 61 in FORS. A complete list of the metabolite numbers used to analyse metabolite overlap among the samples is provided in Supplementary Table S2. This implies that *Rhizopus* spp. rapidly diversify the metabolite profile of okara, potentially leading to more abrupt changes in its nutritional and sensory properties. The greater number and concentration of metabolites in *Rhizopus*-fermented samples also suggest a dynamic metabolic activity that could be further optimised by adjusting the fermentation period (Gao et al., 2025). This is supported by Chen et al. (2023), who reported enhancement in antioxidant activity during the maturation of fermented soybeans with *R. oryzae*.

This study detected only one flavonoid, genistein, which was present in all samples. Soybeans and their derivative products are well-known as rich flavonoid sources, especially of isoflavonoids (Barnes, 2010). We suspect that the deficit in detected flavonoid compounds is due to our extraction method not being optimal for flavonoids. Specifically, extracts were prepared directly from high-moisture fresh samples using absolute methanol, a condition that likely favoured the extraction of highly polar metabolites while reducing the recovery of semi-polar compounds, such as isoflavones. The fresh samples contained ∼60% water, which might be preferred as a solvent by higher-polarity compounds. Soybean isoflavones are generally more efficiently extracted using aqueous methanol or ethanol systems, thereby improving the extraction of semi-polar phenolics from the matrices (Bragagnolo et al., 2022). Nemitz et al. (2017) previously determined that 80% ethanol is well-suited for the analysis of flavonoids in defatted soybean powder. Consequently, the extraction method used here likely enriched polar metabolites while failing to efficiently recover key soybean flavonoids, explaining why typical soy isoflavones were not clearly detected in the LC-MS analysis. Future metabolomic studies on fermented soybean products, such as tempeh, should utilise an optimised solvent system, preferably 70% ethanol (Bragagnolo et al., 2022), to ensure optimal extraction of both polar and semi-polar metabolites. Beyond the extraction method used, it merits mention that the quantity and types of flavonoids available in soybeans largely depend on plant genotype (Miladinović et al., 2019).

**Fig. 5:**
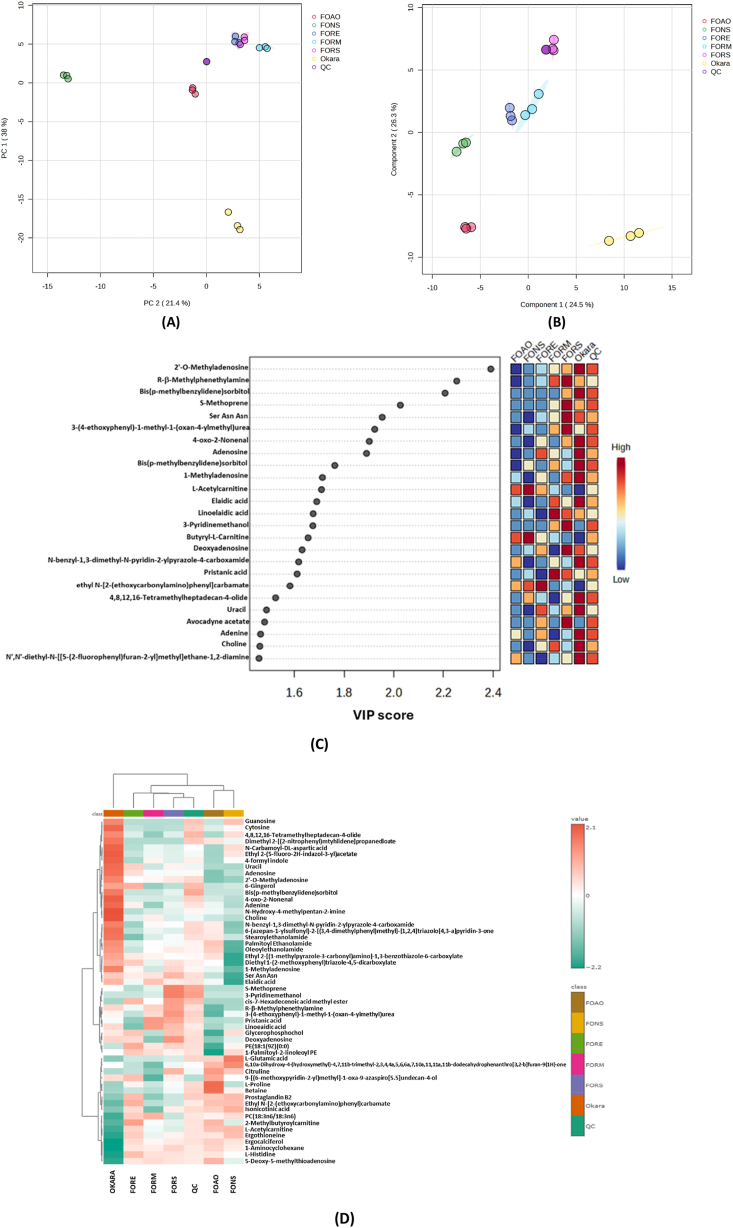
Chemometrics analysis of okara and FFO. (**A**) Principal component analysis; (B) Partial least squares-discriminant analysis; (**C**) The 25 metabolites with the highest VIP scores; (**D**) Combined heatmap and hierarchical clustering analysis. Analysis was conducted using normalised data with auto-scale on https://www.metaboanalyst.ca/.

The effect of fungal starter selection on FFO metabolite profile was evaluated and key differentiating metabolites identified through PCA, PLS-DA, and heatmap clustering analysis. PCA and PLS-DA showed clear separation according to the starter used (Fig. 5A and B), with *Rhizopus* spp. samples (FORE, FORS, and FORM) grouping together and FOAO, FONS, and non-fermented okara all distinctly separated. The robustness of the PLS-DA model was evaluated using cross-validation and permutation testing, as presented in Supplementary Material Table S3 and Fig. S1, respectively. The model with three components showed strong explanatory and predictive power, with *R*^2^ = 0.759 and *Q*^2^ = 0.586, indicating good model fit and predictive ability. To further assess potential overfitting, a permutation test with 1000 random class-label permutations was performed, and it was observed that prediction accuracy was higher than that of the permuted models (*p* < 0.01), confirming that the discrimination among groups was not due to random chance.

A total of 61 differentiating metabolites were selected based on VIP score (VIP >1), of which the top 25 metabolites are presented in Fig. 5C. A heatmap was constructed for the 61 metabolites and combined with hierarchical clustering (Fig. 5D), which grouped the metabolites into two main clusters, separating non-fermented okara from FFO. The FFO cluster was further divided into two, with one cluster consisting of the *Rhizopus* spp. group and the quality control (QC) sample, and the second comprising FOAO and FONS. Notably, the QC sample was prepared by pooling all of the analysed samples in equal amounts. The metabolomic pattern observed in the present study suggests coordinated biochemical activities associated with fungal growth and adaptation during fermentation. Heatmap analysis (Fig. 5D) demonstrates the key metabolites in the present comparative study. The metabolites are choline–betaine transformation, and formation of glutamate, L-proline, linoelaidic acid, pristanic acid, prostaglandin B2, and ergocalciferol.

**Choline**–**betaine transformation**: Among individual metabolites, choline was notably prominent in okara, and was reduced after fermentation with all of the fungi in this study. Choline is essential to preserving structural integrity in all plant and animal cells (Zeisel et al., 2012). Its depletion following fermentation may have been due to fungal enzymatic degradation. The conversion of choline to glycine betaine generally proceeds via a two-step oxidation: choline is first converted to betaine aldehyde by either choline dehydrogenase or choline oxidase, then further oxidised to betaine by betaine aldehyde dehydrogenase (Zou et al., 2016). Although betaine aldehyde was not detected in the present metabolomic dataset, this intermediate product is highly reactive and often remains undetectable due to rapid conversion to glycine betaine. The choline–betaine transformation in FOAO may initiate with the oxidation of choline to betaine aldehyde by choline dehydrogenase rather than choline oxidase. This hypothesis is supported by the detection of betaine aldehyde in metabolomic analysis of *A. oryzae* grown on a nori substrate, which indicates the presence of this pathway in filamentous fungi (Inoue et al., 2025). The accumulation of betaine aldehyde as an intermediate in choline–betaine transformation is considered a marker distinguishing choline dehydrogenase from choline oxidase activity. Although direct enzymatic characterisation in *N. sitophila* and related fermentation systems remains limited, it is plausible that *N. sitophila* employs a similar choline oxidation pathway, although further enzymatic or genomic investigation is required to confirm this. In addition, fungal expression of phospholipases might influence the choline content in FFO by degrading the choline-binding phospholipid in okara (Barman et al., 2018). *A. oryzae* and *N. crassa* have both been shown to be able to produce a certain type of phospholipase whose activity facilitates the synthesis of betaine (Machida et al., 2005; Takayanagi et al., 2015), which we found to be richly abundant in FOAO and FONS. Betaine is widely recognised as a compatible solute that helps microorganisms maintain osmotic balance under environmental stress, suggesting that its accumulation may contribute to fungal adaptation during fermentation (Zou et al., 2016). Interestingly, betaine exhibits multiple biological activities with significant health benefits, particularly in preventing metabolic diseases (Yang et al., 2025).

**Glutamate and L-proline formation:** Another notable metabolite is glutamic acid, the formation of which was observed in FOAO and FONS, likely due to glutaminase production by *A. oryzae* and *Neurospora* sp. (Dantzig et al., 1979; Bazaraa et al., 2016). Glutaminase is one of the central enzymes driving umami formation, fermentation efficiency and overall flavour development in dishes containing fermented soybean, such as soy sauce. Glutaminase is an amidohydrolase enzyme that catalyses the hydrolysis of L-glutamine into L-glutamic acid and ammonia, which directly generates the dominant umami compound in soy sauce (Kijima and Suzuki, 2007). Soybeans contain ∼46% of glutamate in the form of glutamine, which would otherwise degrade into tasteless pyroglutamic acid without glutaminase activity. Glutaminase prevents this loss by redirecting glutamine into glutamate, preserving and enhancing umami flavour (Siwei et al., 2025). In addition, L-proline was only detected in FOAO, and hence may serve as a metabolite marker for soy-based products fermented by *A. oryzae*. Glutamate metabolism in fungi is closely connected to proline biosynthesis through the glutamate–proline cycle. Enzymes such as pyrolline-5-carboxylate (P5C) and proline dehydrogenase mediate the interconversion between glutamate and proline (Kavi Kishor et al., 2022). Additionally, synthesis of L-proline by microorganisms may be facilitated by secreted prolyl aminopeptidases (Dong et al., 2022), which *Aspergillus* fungi have previously been demonstrated capable of producing (Basten et al., 2005; Matsushita‐Morita et al., 2010). Prolyl aminopeptidase is a proteolytic enzyme that cleaves N-terminal proline residues from peptides (Dong et al., 2022). While it does not catalyse proline biosynthesis directly*,* it could increase free proline availability by releasing proline from proteins in okara. Both glutamic acid and L-proline are important in the food industry as flavour enhancers, as they contribute to the umami and sweet tastes, respectively (Schiffman et al., 1981). This observation may highlight the potential for cost-effective production of these amino acids through fermented food processes (i.e. fungal-fermented okara), especially utilising *A. oryzae* and *N. sitophila*.

**Linoelaidic acid, pristanic acid, and prostaglandin B2 formation:** Several lipid derivatives were detected in okara fermented with *Rhizopus* spp.*,* such as pristanic acid and linoelaidic acid in FORM and FORS, and prostaglandin B2 in FORE. These metabolites serve as distinguishing markers for *Rhizopus-*fermented samples compared to those produced with other starters in the present study. They are linked to lipid oxidation, branched-chain fatty acid metabolism, and oxylipin formation (Wanders et al., 2011; Di Dato et al., 2020). Although *Rhizopus* are remarkable for their capability to produce a wide range of lipid-modification enzymes that mediate hydrolysis, interesterification, alcoholysis, and esterification reactions (Yu et al., 2016), the detection of these specific metabolites in *Rhizopus-*fermented soybean has not been previously reported. Their identification in this study highlights previously underexplored aspects of fungal lipid metabolism that may contribute to the distinctive biochemical profile of *Rhizopus*-driven fermentation. Future studies should focus on elucidating these metabolites to better understand their roles and metabolic dynamics.

**Ergocalciferol formation:** In a pattern opposite to choline, ergocalciferol was absent in okara but detected in all of the fungal-fermented samples. Ergocalciferol, also known as Vitamin D2, is generated through the photochemical conversion of ergosterol in fungal cells following exposure to light (Guan et al., 2016). Because ergosterol is a major sterol component of fungal membranes and is likely to accumulate in FFO during fermentation, light-induced transformation may contribute to ergocalciferol accumulation across the FFO. Interestingly, ergocalciferol is reportedly prominent in fungi after continuous exposure to UV light (Guan et al., 2016; Jasinghe and Perera, 2006; Wu and Ahn, 2014). In the present study, ergocalciferol formation may therefore be attributed to uncontrolled light exposure during and after the fermentation process. These observations highlight the potential influence of light conditions on ergocalciferol formation in FFO and indicate that the systematic evaluation of light intensity and exposure time is warranted in future studies to justify ergocalciferol production in fungal-containing products, such as FFO.

Overall, the LC-MS analysis in this work identified choline and ergocalciferol as the primary metabolites distinguishing unfermented and fungal-fermented okara. In addition, metabolites found to be distinctive among the fungal starters were betaine and L-glutamic acid, produced by *A. oryzae* and *N. sitophila*, and some lipid derivative metabolites (pristanic acid, linoelaidic acid), produced by *Rhizopus* spp.

### 3.4 Phenolic content and antioxidant activity evaluation

Phenolic compounds were evaluated in terms of TPC, TFC, and TCTC; the results are provided in Table 2. Fungal fermentation was found to elevate all of the evaluated phenolic contents by 2.19- to 4.13-fold. TPC was assayed with the Folin-Ciocalteu reagent, and found to range from 0.264 to 1.029 mg of gallic acid equivalent (GAE) in fresh samples, with the lowest level occurring in unfermented okara and the highest level in FONS. Development of TPC did not differ significantly among FONS, FORE, and FORS; however, all of the FFO samples were significantly different from the unfermented okara. This result aligns with a previous study of soybean fermented by *R. oligosporus*, in which TPC was increased from 2.55 to 9.28 GAE mg/g (Zhang et al., 2022). Conversely, Polanowska et al. (2020) reported a gradual decrease over time of TPC in faba beans fermented by *R. oligosporus*.

**Table 2:** Total phenolic, flavonoid, and condensed tannin contents and the antioxidants assay results.

Sample	TPC	TFC	TCTC	DPPH	ABTS	FRAP
**Okara**	0.264 ± 0.03^c^	0.113 ± 0.001^c^	0.082 ± 0.01^c^	0.106 ± 0.01^e^	0.241 ± 0.02^c^	0.846 ± 0.06^c^
**FORE**	0.997 ± 0.14^ab^	0.450 ± 0.07^a^	0.250 ± 0.03^a^	0.305 ± 0.02^a^	0.555 ± 0.07^a^	1.268 ± 0.24^abc^
**FORS**	1.008 ± 0.09^a^	0.346 ± 0.04^ab^	0.290 ± 0.01^a^	0.267 ± 0.01^bc^	0.620 ± 0.01^a^	1.555 ± 0.08^ab^
**FORM**	1.090 ± 0.01^a^	0.312 ± 0.04^b^	0.256 ± 0.04^a^	0.245 ± 0.01^cd^	0.328 ± 0.02^c^	1.718 ± 0.55^a^
**FOAO**	0.806 ± 0.03^b^	0.248 ± 0.01^bc^	0.182 ± 0.003^b^	0.231 ± 0.01^d^	0.422 ± 0.03^b^	1.271 ± 0.06^abc^
**FONS**	1.029 ± 0.01^a^	0.462 ± 0.08^a^	0.278 ± 0.01^a^	0.291 ± 0.01^ab^	0.613 ± 0.02^a^	0.984 ± 0.12^bc^

TPC, total phenolic content expressed in mg gallic acid equivalent per g fresh sample (wet basis); TFC, total flavonoid content in mg quercetin equivalent per g fresh sample (wet basis); TCTC, total condensed tannin content in mg catechin equivalent per g fresh sample (wet basis).

ABTS, DPPH, and FRAP assays are expressed in mg ascorbic acid equivalent per g fresh sample (wet basis).

Different letters indicate statistically significant difference in means within a column based on Tukey's HSD at the 95% confidence level.

TFC was evaluated based on formation of flavonoid-Al complexes, and expressed in mg of quercetin equivalent per g fresh sample. TFC was drastically increased after fungal fermentation (*p* < 0.05), with the highest concentration obtained for FORE, followed by FONS, FORS, FORM, and then FOAO. This result is in contrast to a prior study by Liu et al. (2023), which showed a decrease of TFC in fermented soybean during tempeh production. However, the researchers also observed specific development of flavonoid aglycons, such as daidzein, genistein, and glycitein, during the fermentation process. Alteration of flavonoid glucosides into flavonoid aglycons is carried out by β-glucosidase (Delmonte and Rader, 2006), which is known to be produced by *Rhizopus, Aspergillus,* and *Neurospora* (Farniga et al., 2023; Zhang et al., 2022; Wu et al., 2013).

Although increased TPC and TFC were observed after fungal fermentation, untargeted LC-MS analysis revealed a limited number of phenolic compounds, especially flavonoids. It should be highlighted that the Folin-Ciocalteu and aluminium chloride reagents react with any reducing substances and metal-chelating compounds, respectively, not just phenolic compounds, including amino acids, nucleosides, vitamins, and other redox-active metabolites (Rothwell et al., 2013), which are abundantly detected in this study, and therefore these assays can overestimate TPC and TFC. Moreover, extraction from a fresh sample with a high-moisture matrix using absolute methanol may preferentially enrich highly polar metabolites while suppressing semi-polar isoflavones during LC-MS analysis, explaining why typical isoflavones were not clearly detected in the LC-MS dataset despite the observed increase in TPC and TFC. A comprehensive metabolite profiling study of soy by-products identified an optimised extraction method using 70% ethanol (Barnes, 2010), yielding the greatest number of detected flavones, including isoflavonoids. Furthermore, Kim and Kim (2025) reports a substantial number of identified phenolic compounds, particularly flavonoids, in their metabolomics study of dried peach extracts obtained using 99.9% methanol with ultrasonic assistance. In addition to extraction methods, extending the LC-MS run time could increase the number of detected phenolic compounds, as the current run time was limited to 15 min. In the metabolite profiling of soy by-products, several isoflavonoids (e.g., genistein and daidzein) were detected after 15 min in their LC-MS system (Bragagnolo et al., 2022).

TCTC was determined based on the reaction between condensed tannin with acidified vanillin and expressed in mg catechin equivalent (CE) per g fresh sample. Overall, the results showed a trend similar to TPC and TFC analysis, with elevation of TCTC in FFO samples compared to okara. The highest TCTC was obtained for FORS, with 0.290 mg CE per g fresh sample (2.88 times greater than okara). Among FFO samples, FOAO exhibited the least TCTC, TFC, and TPC, but its phenolic compound content was still much greater than okara (*p* < 0.05). The elevation of TCTC following fermentation might be attributable to synthesis of flavonols, which compound class is the most reactive in the acidified vanillin assay (Broadhurst and Jones, 1978).

Antioxidant content was likewise investigated through ABTS, DPPH, and FRAP assays, with the results expressed in mg ascorbic acid equivalent per g fresh sample. The ABTS and DPPH assays determine the ability of antioxidant compounds to scavenge free radicals, while the FRAP assay estimates antioxidant reducing power through reduction of Fe^3+^ into Fe^2+^ in acidic conditions (Čeryová et al., 2026). Table 2 presents the antioxidant properties of the tested samples. The results are consistent with multiple previous reports of antioxidant activity enhancement after fungal fermentation of soybean (Cai et al., 2024; Huang et al., 2021; Asni et al., 2024). While ascorbic acid was used as a reference antioxidant, it serves as a simple, water-soluble electron donor and does not fully represent the complex antioxidant mechanisms of metabolites derived from fermentation, such as polysaccharides, lipids, and other non-phenolic compounds (Abramovič et al., 2018; Wołosiak et al., 2021).

Untargeted metabolomic profiling indicated that several metabolites, including betaine, carnitine, ergocalciferol, linoelaidic acid, and pristanic acid, were upregulated in the fermented samples. While these compounds are reported to exhibit antioxidant functions in biological systems, primarily through roles in osmoprotection (Heidari et al., 2018), redox homeostasis (Siervo et al., 2024), or metabolic regulation, their mechanisms do not directly involve free radical scavenging, which underpins assays such as ABTS, DPPH, and FRAP. Therefore, the metabolites detected by LC-MS likely contribute minimally to the antioxidant capacity measured by these specific chemical assays. This distinction suggests that the increased antioxidant activity observed in these assays is more closely associated with phenolic compounds, as reflected in the TPC and TFC, which are recognised for their strong radical-scavenging properties (Čeryová et al., 2026).

**Fig. 6:**
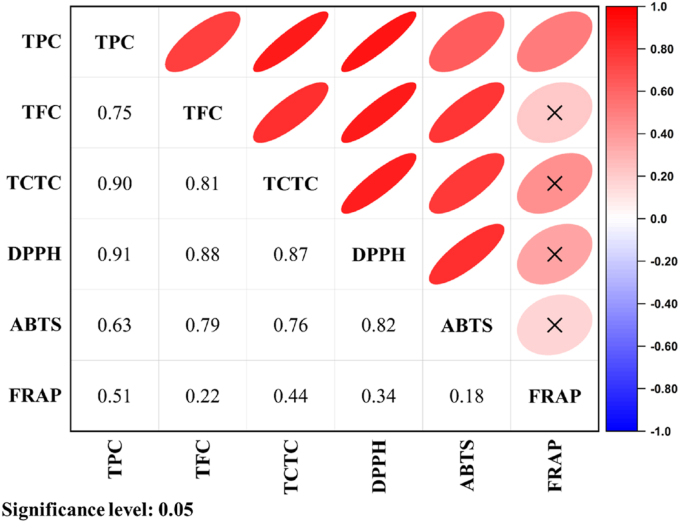
Pearson correlations of the phenolic contents and antioxidant properties of okara and FFO. Cross mark ( × ) indicates insignificant correlation factors at *p* < 0.05.

We also performed Pearson correlation analysis to evaluate the relationship between phenolic content and antioxidant properties. TPC, TFC, and TCTC were all observed to positively correlate with the development of antioxidant activity (Fig. 6). TPC was the only factor influencing FRAP development, with an *R*^2^ value of 0.51 (*p* < 0.05). The observed increase in TPC, TFC, and TCTC across all FFO indicates that each contributes uniquely to enhancing antioxidant properties. This effect is likely due to the utilised fungi's ability to produce hydrolytic enzymes (i.e. β-glucosidase, cellulase, and protease), as this group of enzymes has previously been reported to enhance TPC, TFC, and TCTC, thereby increasing the antioxidant properties in *Eurotium cristatum* fermentation (Fan et al., 2025; Xiao et al., 2021). Collectively, these findings indicate that future research should systematically compare the effects of extraction methods, sample moisture levels, and LC-MS parameters on the diversity of phenolic compounds in fermented soy extracts, especially tempeh.

## 4 Conclusion

This study provides a comprehensive comparative analysis of the metabolite profile of okara before and after fermentation by five different fungal starters. Our findings demonstrate that the choice of fungal starter influences the metabolite composition of the product, highlighting the potential to tailor fermented okara with specific functional properties through strategic selection of fermenting microorganisms. Fermentation with *N. sitophila* notably enhanced glucan content, protein level, and antioxidant properties, indicating its potency for developing nutrient-rich food products. Additionally, *N. sitophila* and *A. oryzae* both promoted the accumulation of betaine and L-glutamic acid, compounds with potential applications in food flavouring. Finally, fermentation with *Rhizopus* spp. induced notable changes in lipid derivatives, which may be relevant for food processing applications.

## CRediT authorship contribution statement

Analdi Farniga: Conceptualization, Data curation, Formal analysis, Methodology, Validation, Visualization, Investigation, Writing – original draft. Praphan Pinsirodom: Conceptualization, Methodology, Investigation. Songsak Wattanachaisaereekul: Conceptualization, Data curation, Methodology, Validation, Investigation, Supervision, Writing – review & editing, Project administration.

## Funding

The authors are grateful for the financial support provided by King Mongkut's Institute of Technology Ladkrabang Research Fund (Grant number: 2564-02-07-001).

## Declaration of competing interest

The authors declare that they have no known competing financial interests or personal relationships that could have appeared to influence the work reported in this paper.

## Data Availability

Data will be made available on request.
